# Equilibrium among Inflammatory Factors Determines Human MSC-Mediated Immunosuppressive Effect

**DOI:** 10.3390/cells11071210

**Published:** 2022-04-03

**Authors:** Yulia Suzdaltseva, Kirill Goryunov, Ekaterina Silina, Natalia Manturova, Victor Stupin, Sergey L. Kiselev

**Affiliations:** 1 Department of Epigenetics, Vavilov Institute of General Genetics, Russian Academy of Sciences, 119333 Moscow, Russia; sl_kiselev@yahoo.com; 2 Department of Cell Technologies, National Medical Research Center for Obstetrics, Gynecology and Perinatology, 117997 Moscow, Russia; kirishgor@gmail.com; 3 Institute of Biodesign and Modeling of Complex Systems, I.M. Sechenov First Moscow State Medical University, 119991 Moscow, Russia; silinaekaterina@mail.ru; 4 Department of Plastic and Reconstructive surgery, Cosmetology and Cell Technologies, Pirogov Russian National Research Medical University, 117997 Moscow, Russia; manturovanatali@yandex.ru; 5 Department of Hospital Surgery, Pirogov Russian National Research Medical University, 117997 Moscow, Russia; stvictor@bk.ru

**Keywords:** immunomodulation, mesenchymal stem cells, cytokines, priming

## Abstract

Mesenchymal stem cells (MSCs) are thought to be a promising therapeutic agent due to their multiple paracrine and immunomodulatory properties, providing protection from chronic inflammation and promoting tissue repair. MSCs can regulate the balance of pro-inflammatory and anti-inflammatory factors in inflamed tissues, creating a microenvironment necessary for successful healing; however, their interactions with immune cells are still poorly studied. We examined the temporal and spatial changes in gene regulation and the paracrine milieu accompanying the MSC-mediated immunosuppression effect in mixed cultures with activated peripheral blood mononuclear cells (PBMCs). Our data reveal that the peak of suppression of PBMC proliferation was achieved within 48 h following co-culture with MSCs and subsequently did not undergo a significant change. This effect was accompanied by an increase in COX-2 expression and an induction of IDO synthesis in MSCs. At this point, the expression of IL-1, IL-6, IL-8, IFN-γ, MCP-1, and G-CSF was upregulated in co-cultured cells. On the contrary, we observed a decrease in the concentrations of IL-10, IL-13, IL-5, and MIP-1b in co-culture supernatants compared to intact cultures of activated PBMCs. The regulation of IDO, IL-1, IL-6, and G-CSF production was accomplished with the involvement of direct cell–cell contact between MSCs and PBMCs. These findings provide new insights into the use of potential precondition inducers or their combinations to obtain functionally qualified MSCs for more effective treatment of inflammatory diseases.

## 1. Introduction

Mesenchymal stem cells (MSCs) are multipotent cells that are able to regulate tissue regeneration due to their multiple paracrine and immunomodulatory effects. MSCs are currently considered to be a promising therapeutic agent, since they can be readily isolated from bone marrow and adipose tissue and successfully expanded in vitro. The potent mechanisms of MSC-based therapy are the stimulation of neovascularization and immunomodulatory activity, reduction of fibrosis, and the stimulation of endogenous tissue regeneration acting in concert [1,2]. The cytokines, chemokines, growth factors, and extracellular vesicles secreted by MSCs are involved in the regulation of intracellular signaling cascades that stimulate angiogenesis, the recruitment of cells to sites of inflammation, and remodeling of the extracellular matrix [3].

External signals from the microenvironment alter the expression and secretory profile of MSCs, allowing them to perform a regulatory function at the site of inflammation [4]. During early inflammatory response following specific stimulation through receptors, MCSs may be polarized toward functionally different phenotypes [5]. Therefore, the local milieu is crucial for inducing the functional activity of MSCs.

MSCs have been shown to exhibit immunomodulatory effects when interacting with immune cells, and particularly to inhibit T cell proliferation in mixed lymphocyte reactions [6]. They also suppress the differentiation and maturation of dendritic cells (DCs) and promote their polarization toward the anti-inflammatory M2 phenotype [7]. MSCs mediate the induction of a regulatory phenotype in conventional T cells (Tregs) [8,9]. MSCs can induce functional changes in immune cells, and thus have the ability to regulate the balance between pro-inflammatory and anti-inflammatory factors produced by them, creating a cytokine microenvironment that is necessary for successful healing [10,11]. This immunomodulatory activity of MSCs may control the course of inflammation and its transition to subsequent phases of tissue repair.

It is now accepted that MSCs exert pleiotropic therapeutic effects mainly via the secretion of soluble factors. Experimental data from a large number of in vitro and preclinical studies have demonstrated that MSC-derived conditioned medium (CM) exhibits similar effects to those observed after the transplantation of MSCs [12]. Previous in vitro studies showed that the secretome of cultured MSCs includes cytokines, growth factors, chemokines, extracellular matrix proteins, and metalloproteinases. Within the MSC secretome several growth factors were identified, including VEGF, TGF-β1, TGF-β2, HGF, bFGF, IGF2, PDGF, FGF7, GM-CSF, and G-CSF, which are known to be involved in cell communication and signal transduction. MSCs have also been shown to produce chemokines MCP-1, MIP-1α, RANTES, and IP-10, as well as cytokines IFN-γ, IL-1, IL-4, IL-6, IL-8, IL-12, IL-15, and IL-17, the combined reaction of which underlies the immunomodulatory effects of MSCs [13,14,15]. Paracrine factors secreted by MSCs perform anti-inflammatory, immunomodulatory, chemoattractive, anti-apoptotic, anti-oxidative, and anti-fibrotic functions in inflamed tissues, promoting the healing process [16,17]. However, the temporal and spatial changes in the paracrine milieu that occur under the influence of MSCs during inflammation are poorly understood.

The present study was aimed at evaluating alterations in the expression and secretory profiles of human adipose-derived MSCs under the influence of external signals from the pro-inflammatory microenvironment produced by activated peripheral blood mononuclear cells (PBMCs) and accompanying the effect of suppressing their proliferation. For this purpose, separate cultures of MSCs and PBMCs and mixed direct or non-contact co-cultures were used. Adipose-derived MCS were chosen for the study because they are more easily accessible and show a strong capacity for ex vivo expansion without ethical concerns. Qualitative and quantitative analyses of proteins relevant to inflammation and immune regulation were performed in MSCs and supernatants of separate and mixed cultures of MSCs and PBMCs.

## 2. Materials and Methods

### 2.1. Isolation and Culture of Human Adipose-Derived Mesenchymal Stromal Cells

Specimens of human subcutaneous adipose tissue were collected as waste material, which was a byproduct of elective surgery at the Department of Surgery of Municipal Clinical Hospital No. 15 (Moscow, Russia) from patients who signed voluntary informed consent forms under the intramural scientific agreement. Isolation and cultivation of MSCs were performed using the enzymatic method, as previously described [18]. Briefly, the adipose tissue samples were washed extensively with Dulbecco’s phosphate-buffered saline (DPBS, HyClone, Cytiva, Marlborough, MA, USA) and minced into small pieces, which then underwent enzymatic digestion in type I collagenase enzyme (200 U/mL; Worthington Biochemical, Lakewood, NY, USA) and dispase (40 U/mL; Sigma-Aldrich, St. Louis, MO, USA) at 37 °C for 30–45 min. Then an equal volume of Dulbecco’s Modified Eagle Medium/Nutrient Mixture F-12 (DMEM/F12; Thermo Fisher Scientific, Waltham, MA, USA), supplemented with 100 U/mL penicillin and streptomycin, 2 mM L-glutamine (HyClone, USA), and 10% fetal bovine serum (One Shot FBS; Gibco, Thermo Fisher Scientific, USA), was added. After centrifugation at 260 g for 5 min, the pellets were resuspended in culture medium and plated in Petri dishes (Corning, Corning, NY, USA). The cells were cultured in a humidified atmosphere containing 5% CO_2_ at 37 °C until ~70%–80% confluence. The medium was changed every 3–4 days. Cells at passage 3 and 4 were used for experiments.

### 2.2. Verification of MSC Identity

Immunocytochemical staining of culture-expanded MSCs as surface markers of mesenchymal stromal and hemopoietic cells was performed with antibodies to CD105, CD73, CD90, CD34, CD45, and CD14 conjugated with fluorochromes Alexa Fluor 647, allophycocyanin (APC), and pyridine chlorophyll–cyanine5.5 (Per-CP-Cy5.5) (BD Bioscience, San Jose, CA, USA) according to the manufacturer’s instructions. Samples were analyzed using a FACSCanto II flow cytometer (BD Biosciences, San Jose, CA, USA). The data were processed using the FlowJo software package. The ability of UCMSCs to differentiate into adipogenic, osteogenic, and chondrogenic lineages was evaluated, as described previously [18]. To induce adipogenic differentiation MSCs were cultured for 2–3 weeks in DMEM/F12 supplemented with 10% equine serum, 0.5 μM hydrocortisone, 0.5 mM isobutylmethylxanthine, 60 μM indomethacin, 100 U/mL penicillin, 100 U/mL streptomycin, 2 mM glutamine, 1 mM sodium pyruvate, replaced weekly. Before and after culture, cells were stained with fresh Oil-Red O solution (Sigma-Aldrich, St. Louis, MO, USA). Osteogenic differentiation was performed by culturing the cells with serum-free DMEM/F12 supplemented with 0.2 mM ascorbate, 10 mM β-glycerophosphate, 10–7 M dexamethasone, 100 U/mL penicillin, 100 U/mL streptomycin, 2 mM glutamine, and 1 mM sodium pyruvate (Sigma-Aldrich, St. Louis, MO, USA ) for 2–3 weeks. The medium was replaced weekly. To assess calcium accumulation, cultures were stained with Alizarin Red (Sigma-Aldrich, St. Louis, MO, USA) and scored for areas of mineralization. MSC micromass pellets were exposed to DMEM/F12 supplemented with 10% FBS, 100 ng/mL TGF-β (Sigma-Aldrich, St. Louis, MO, USA) for 2–3 weeks for chondrogenic differentiation. After 2 weeks in culture, cryosections of micromasses were stained with 1% toluidine blue in 50% isopropanol.

### 2.3. Isolation and Activation of Human PBMCs

Whole blood was donated by healthy donors who were negative for infectious markers (hepatitis B and C, HIV-1 and 2, and *Treponema pallidum*) after providing informed consent. PBMCs were isolated from buffy-coats of whole blood via centrifugation on Histopaque density gradient (ρ = 1.077 g/cm^3^; Sigma Aldrich, St. Louis, MO, USA). Then the PBMCs were washed in DPBS and resuspended in culture medium. The PBMCs were activated by phytohemagglutinin (PHA; Sigma Aldrich, St. Louis, MO, USA) at a concentration of 10 μg/mL for 2 h at 37 °C. Then the suspension was centrifuged and the pelleted cells were resuspended in RPMI-1640 medium (Sigma-Aldrich, St. Louis, MO, USA) supplemented with 100 U/mL penicillin and streptomycin and 10% FBS to a concentration of 1 × 10^7^ cells/mL.

### 2.4. PBMC Proliferation Assay

MSCs were seeded in 12-well plates at an initial density of 1.2 × 10^3^ cells/cm^2^ and cultured for 48 h before the co-culture experiment. The supernatant was then collected and replaced with RPMI-1640 medium supplemented with 10% FBS. PHA-activated PBMCs were added to MSCs to achieve ratios of 10:1, 25:1, 50:1, and 100:1. Mixed MSCs and PBMCs were cultured in contact or in a Transwell system with a 0.4 μm pore size membrane (Greiner Bio-One, Kremsmünster, Austria) at 37 °C and 5% CO_2_ for 24–96 h. MSC conditioned medium (MSC CM) and RPMI-1640 medium supplemented with 10% FBS were used as controls for separate PBMC cultures. Proliferation of PBMCs was assessed via the quantitation of fluorescence intensity using the CyQUANT^®^ NF Cell Proliferation Assay Kit (Thermo Fisher Scientific, Waltham, MA, USA). Non-adherent PBMCs were harvested from the supernatant via gentle pipetting. Then 50 μL of each PBMC sample was transferred in duplicate to a 96-well black FLUOTRAC plate (Greiner Bio-One, Kremsmünster, Austria) and mixed with an equal volume of CyQUANT^®^ NF reagent. Fluorescence intensity was measured at 530 nm using a Zenyth 3100 spectrophotometer (Anthos, Biochrom, Cambridge, UK). The cell proliferation rate was determined by normalizing the fluorescence intensity of the samples to that of activated PBMCs cultured in RPMI-1640 medium.

### 2.5. Cell Pre-Labeling with Calcein

For co-culture experiments, MSCs and PBMCs were labeled with fluorescent dyes. MSC monolayers were stained with CellTrace™ calcein green AM, whereas PBMC pellets were stained with CellTrace™ Calcein red-orange AM (Thermo Fisher Scientific, Waltham, MA, USA) in serum-free DMEM for 30 min at 37 °C in the dark, and subsequently washed twice in PBS. Cells were imaged using an inverted Zeiss Axiovert 200 microscope (Zeiss, Oberkochen, Germany).

### 2.6. Bio-Plex Cytokine Assay

The CM from monocultures of MSCs and activated PBMCs or functional assays of PBMCs was collected at 48 h and centrifuged at 260 g for 10 min, filtered through a 0.22 µm porous membrane (Millipore, Burlington, MA, USA), and immediately stored at −70 °C. A panel of cytokines, chemokines, and growth factors was measured in the CM of separate MSC and PBMC cultures and their co-cultures via an immune-based protein array. A Bio-Plex Pro Human Cytokine 17-plex kit (Bio-Rad, Hercules, CA, USA) was used to measure the concentration of 11 cytokines (IL-1b, IL-2, IL-4, IL-5, IL-6, IL-7, IL-8, IL-10, IL-12(p70), IL-13, IL-17, IFN-γ, and TNF-α), 2 growth factors (G-CSF and GM-CSF), and 2 chemokines (MCP-1 and MIP-1). Quantitative measurements (in duplicate) were performed according to manufacturer’s guidelines using the Luminex Bio-Plex 200 system (Bio-Rad, Hercules, CA, USA) operated using the Bio-Plex Manager™ 6.0 software package. The cell culture medium was used to normalize the measured expression of cytokines and growth factors in CM.

### 2.7. Quantitative PCR Analysis

To evaluate gene expression, total RNA was extracted from MSCs after functional assays with PBMCs using TRIzol reagent (Thermo Fisher Scientific, Waltham, MA, USA) and purified using the phenol/chloroform technique. RNA concentration was quantified using a NanoDrop ND-2000c (Thermo Fisher Scientific, Waltham, MA, USA). Complementary DNA was synthesized from each RNA template using the SuperScript III First-Strand Synthesis System (Thermo Fisher Scientific, Waltham, MA, USA). The expression of IDO, COX-2, and iNOS genes was analyzed using primers designed with the NCBI Primer Designing Tool. Primer sequences can be provided upon request. qPCR reactions were performed using Maxima SYBR Green/ROX qPCR Master Mix (Thermo Fisher Scientific, Waltham, MA, USA) on a DTprime Realtime Detection Thermal Cycler (DNA Technology, Moscow, Russia). Relative gene expression was calculated using the ΔΔCt method with GAPDH or ACTB as the reference gene. The following primers were used: human IDO1: forward AGCCCCTGACTTATGAGAACATGGA, reverse CCAGCCAGACAAATATATGCGAAGAA; human NOS2: forward CTCAGCTGTGCATCGACC, reverse GCCCATGTACCAGCCATTGA; human PTGS2: forward TTCACGCATCAGTTTTTCAAGACAGA, reverse CATCAGACCAGGCACCAGACCA, human ACTB: forward CTCGCCTTTGCCGATCC, reverse TCTCCATGTCGTCCCAGTTG.

### 2.8. Statistical Analysis

Statistical analyses were performed using SPSS 14.0 software (SPSS Inc., Chicago, IL, USA). Statistical comparisons of two datasets were performed using Student’s *t*-test. Data were obtained from at least three independent experiments. Measurement data were represented as mean ± standard deviation. Statistical differences were considered significant at *p* < 0.05. 

## 3. Results

### 3.1. Verification of MSC Identity

**Figure 1:**
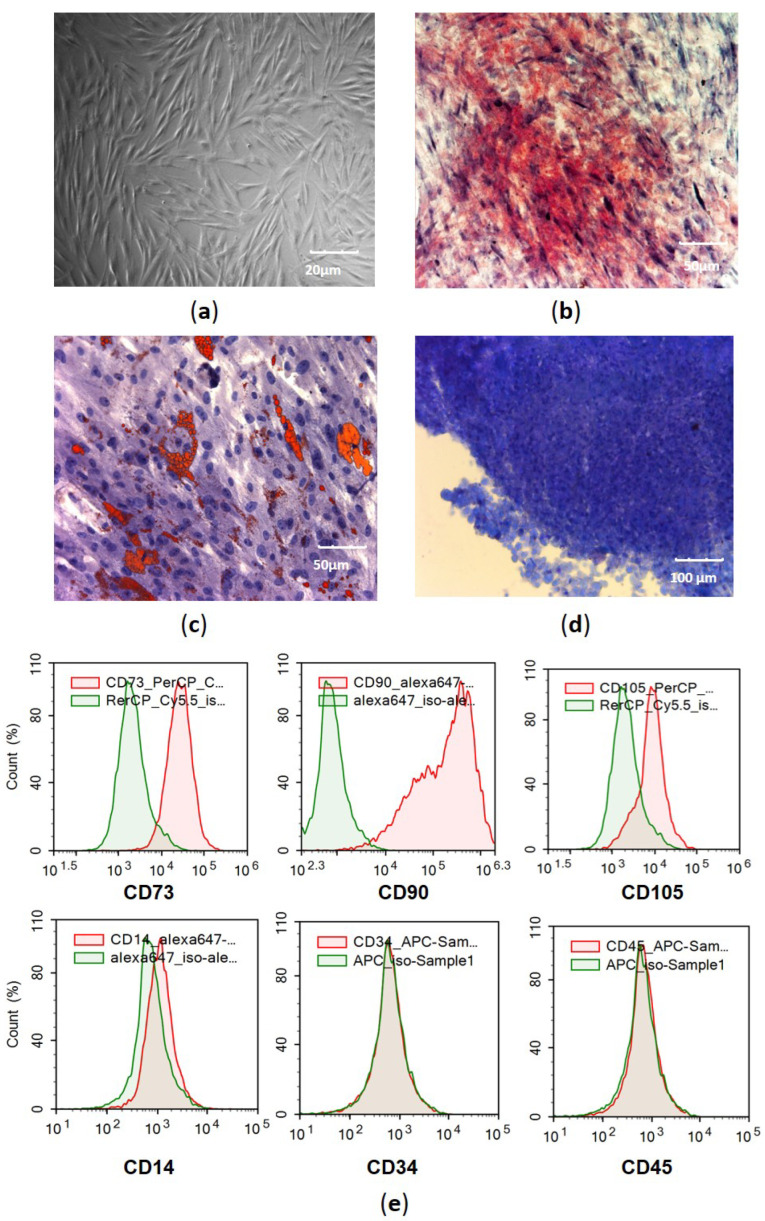
Morphology, phenotype, and multilineage differentiation potential of adipose-derived MSCs. (**a**) Phase contrast image shows fibroblast-like morphology of MSCs from adhesion culture of second passage; (**b**) accumulation of mineralized matrix in MSCs, with alizarin red staining; (**c**) accumulation of adipose vacuoles in MSCs, with Oil Red O and hematoxylin staining; (**d**) toluidine blue staining of mucopolysaccharide extracellular matrix in cryosections from MSC micromass pellets cultured in chondrogenic media; and (**e**) flow fluorometry of MSCs stained with antibodies to surface markers CD90, CD73, CD105 CD34, CD45, and CD14 conjugated with Alexa Fluor 647, APC, and Per-CP-Cy5.5, with green indicating isotype control and red indicating specific surface markers.

The identity of expanded MSCs was confirmed using the minimal criteria proposed by the ISCT [19]. Fibroblast-like MSCs from passage 3 attached to the plastic surface were positive for CD73, CD90, and CD105 expression and negative for CD34, CD45, and CD14 expression. These cells were able to differentiate into adipogenic, osteogenic, and chondrogenic lineages under special conditions (Figure 1).

### 3.2. MSCs Inhibit Proliferation of Activated PBMCs

**Figure 2:**
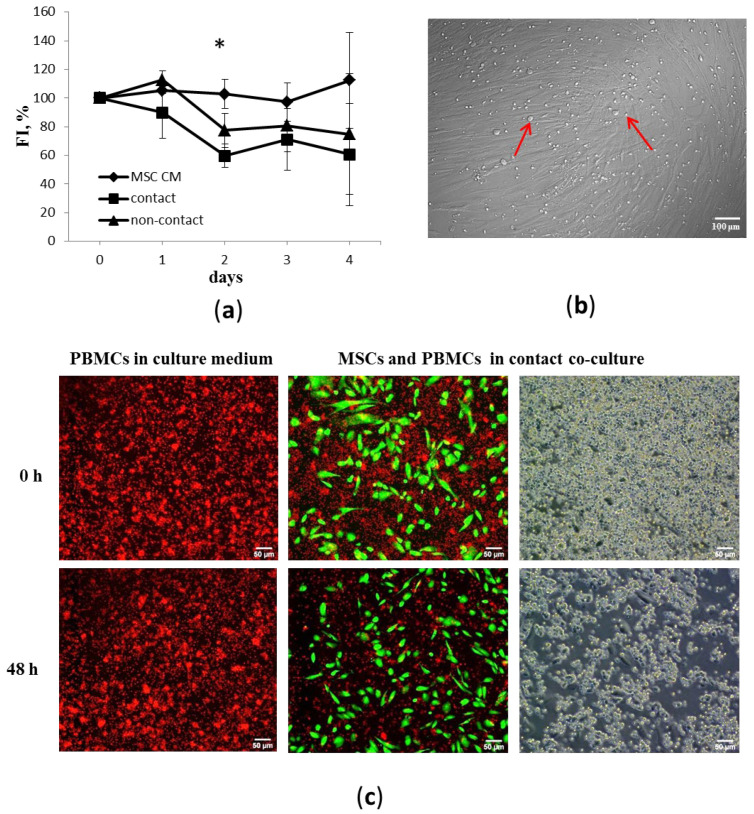
Effects of MSCs on PBMC proliferation. (**a**) Dynamics of PHA-induced proliferation of PBMCs incubated with MSC conditioned medium (MSC CM) or adherent MSCs in contact or non-contact co-cultures at a 25:1 ratio, respectively. Five unrelated donors per cell type were used. Average values and standard errors from five independent fluorescence intensity measurements using CyQUANT^®^ NF (FI) are shown. Data are normalized to the level of proliferation (100%) of PBMCs cultured in RPMI-1640 medium. Asterisk (*) indicates the reliability of differences at *p* < 0.05. (**b**) Red arrows indicate immune cells that formed close contacts with MSCs. Representative images of MSC and PBMC co-cultures after removing supernatant with nonadherent immune cells, phase contrast. (**c**) Representative images of live cells showing suppressive effect of MSCs (green) on PBMC proliferation (red) (CellTrace ™ calcein green and red-orange AM staining).

The immunomodulatory activity of MSCs was assessed based on their ability to suppress PHA-induced proliferation of allogeneic PBMCs in a mixed-culture system. MSCs were able to suppress PBMC proliferation at PBMC:MSC ratios lower than 25:1. No significant inhibitory activity of MSCs was detected at higher PBMC:MSC ratios (data not shown). An inhibitory effect was observed in both contact and non-contact co-cultures. The most prominent suppressive effect of MSCs on PBMC proliferation was observed after 48 h of co-culture; then there were no further significant changes. The supernatant of intact cultures of MSCs did not affect the proliferation of activated PBMCs (Figure 2). These results confirm the capacity of MSCs to modulate PBMC proliferation through paracrine mechanisms in response to an inflammatory microenvironment. However, the fact that some immunocompetent cells were able to interact directly with MSCs in contact co-cultures indicates that cell–cell communication may also be involved in the immunomodulatory effect (Figure 2b).

### 3.3. Activated PBMCs Modulate Expression of Inflammatory Mediators in MSCs

The enzymes indoleamine 2,3-dioxygenase (IDO), cyclooxygenase 2 (COX-2), and inducible nitric oxide synthase (iNOS), which are the main inflammatory mediators, can affect the functional properties of activated PBMCs. To assess the influence of the inflammatory microenvironment on the paracrine activity of MSCs, we analyzed the gene expression of immunomodulatory mediators COX-2, IDO, and iNOS in MSCs after 48 h co-culture with activated PBMCs. The experimental conditions were the same as in the PBMC proliferation assay.

**Figure 3:**
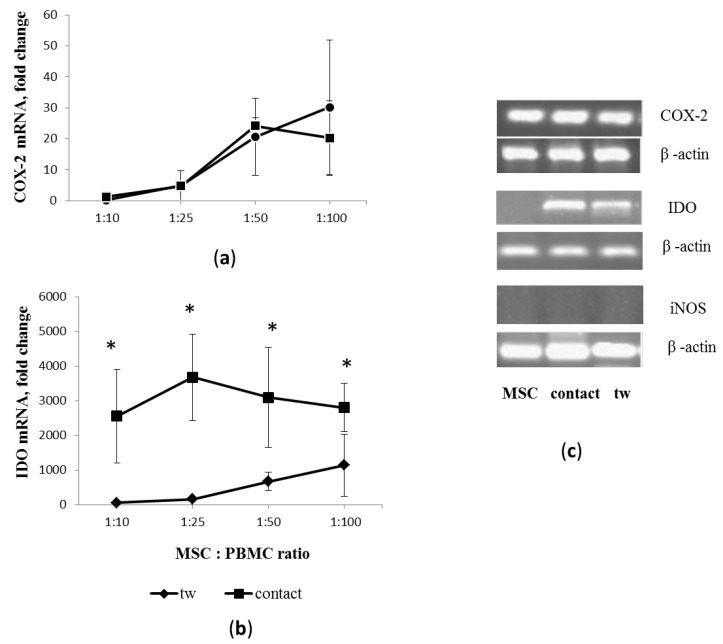
COX-2, IDO, and iNOS expression in MSCs in contact and non-contact (tw) co-culture with PHA-activated PBMCs. (**a**,**b**) Relative changes in COX-2 and IDO expression; mean values and their errors from 3 independent experiments are presented. MSCs were isolated from 3 unrelated donors. * reliability of differences at *p* < 0.05. (**c**) Representative electropherograms of PCR products.

Since COX-2 is a critical enzyme in the prostaglandin synthesis pathway, we assessed the expression level of the COX-2 gene in MSCs when co-cultured with PBMCs. We found that COX-2 mRNA was expressed in intact MSCs constitutively. The COX2 mRNA levels in co-cultured MSCs increased in a dose-dependent manner that was proportional to the number of PBMCs, regardless of the presence or absence of contacts with target cells (Figure 3a).

It was previously reported that the exposure of MSCs to proinflammatory stimuli increases their immunomodulatory function by inducing IDO expression [20]. We found that contact and non-contact co-cultures with PBMCs were responsible for the modulation of IDO synthesis in MSCs. MSCs did not constitutively express the IDO-1 gene. IDO-1 transcripts were undetectable in MSCs from intact cultures. However, induction of IDO-1 gene expression in MSCs was observed during co-culture with activated PBMCs. The expression level of IDO-1 in MSCs was higher in contact than non-contact co-culture with PBMCs. In non-contact co-culture, IDO-1 transcription was elevated in MCSs in a dose-dependent manner with increased PBMC ratios. On the contrary, in contact co-culture, the level of IDO-1 expression in MSCs did not significantly differ depending on the number of PBMCs (Figure 3b).

Studies have shown that the immunosuppressive effects of mouse MSCs occur through the secretion of iNOS (the enzyme responsible for secreting nitric oxide, NO) [21]. Here we demonstrate that in our experimental co-cultures, PBMCs did not trigger iNOS expression in MSCs (Figure 3c).

### 3.4. Multiplex Analysis of Cytokines and Growth Factors in Supernatants of MSC and Activated PBMC Cultures

To further investigate the immunomodulatory effects of MSCs, we analyzed cytokines, chemokines, and growth factors involved in inflammation and immunoregulation in CM derived from intact and mixed non-contact and contact cultures of MSCs and activated PBMCs using a Bio-Plex Human 17-plex assay (Bio-Rad, Irvine, CA, USA).

**Figure 4:**
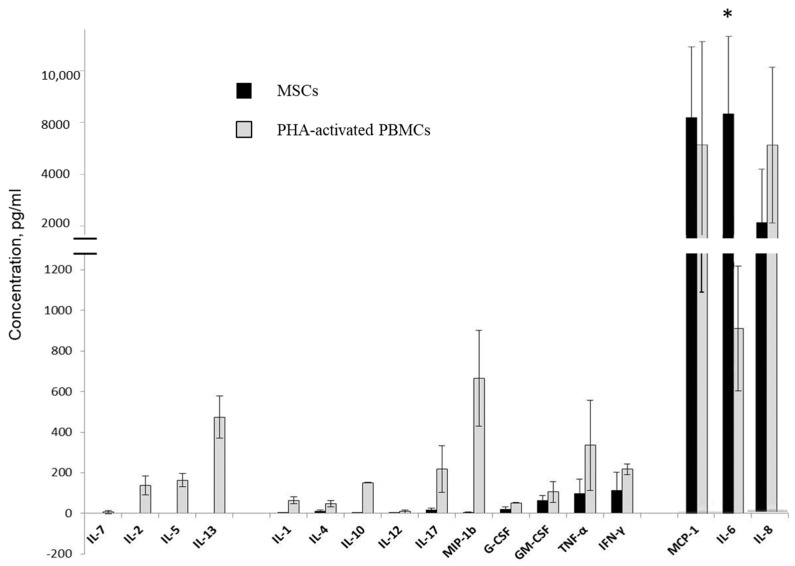
Evaluation of human cytokine concentration in conditioned medium of MSCs and PHA-activated PBMCs, determined via multiplexed fluorescent bead-based immunoassay detection. Three unrelated donors per cell type were used. Data are shown as mean ± std; *t*-test, * reliability of differences at *p* < 0.05.

The supernatants of MSC and activated PBMC cultures from three unrelated donors per cell type were collected after 48 h of culture. The initial seeding density was the same as in the PBMC proliferation assay: 2 × 10^3^ cells/cm^2^ for MSCs and 2 × 10^5^ cells/mL for activated PBMCs. The measured concentrations of cytokines and growth factors in the supernatants were normalized to those in cell culture medium. Resting MSCs secreted IL-1, IL-4, IL-6, IL-8, IL-10, IL-12, IL-17, G-CSF, GM-CSF, IFN-γ, MCP-1, MIP-1b, and TNF-α, whereas IL-2, IL-5, IL-7, and IL-13 were not detected in the supernatants. Activated PBMCs secreted all 17 of the above cytokines. Pro-inflammatory interleukins IL-6, IL-8, and chemokine MCP-1 were most represented in CM of separate MSCs and activated PBMC cultures. The basal level of IL-6 secretion was significantly higher in resting MSCs compared to activated PBMCs. Similar levels of TNF-α, IFN-γ, and GM-CSF secretion were detected in MSCs and activated PBMCs (Figure 4).

### 3.5. Shift of Cytokine Production in MSC and Activated PBMC Co-Cultures

**Figure 5:**
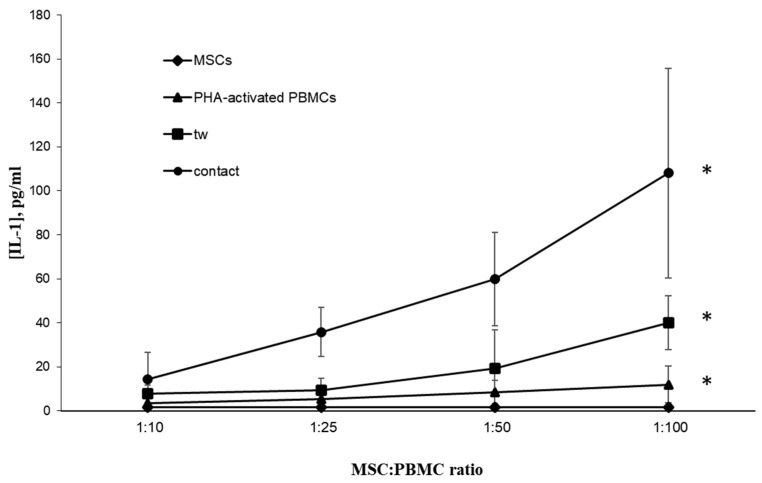
IL-1 concentration in conditioned media of separate MSC and activated PBMC cultures or contact and noncontact (tw) co-cultures. Data are shown as mean ± std; *t*-test, * reliability of differences at *p* < 0.05. Three unrelated donors per cell type were used.

The dose/effect analysis of PBMCs on MSCs, in which the amount of MSCs was constant and the amount of activated PBMCs was increased, revealed that the expression levels of enzyme genes in MSCs and the concentrations of cytokines in CM exhibited a direct proportional relationship with increasing numbers of activated PBMCs. Using the example of IL-1, the effect of PBMC number on the concentration of cytokines in CM is shown in Figure 5. All further measurements were thus performed at an initial seeding density of 2 × 10^3^ cells/cm^2^ for MSCs and 2 × 10^5^ cells/cm^2^ for activated PBMCs in mixed cultures. We also analyzed the CM of supernatants derived from separate cultures of MSCs and activated PBMCs with identical density, which served as controls.

**Figure 6:**
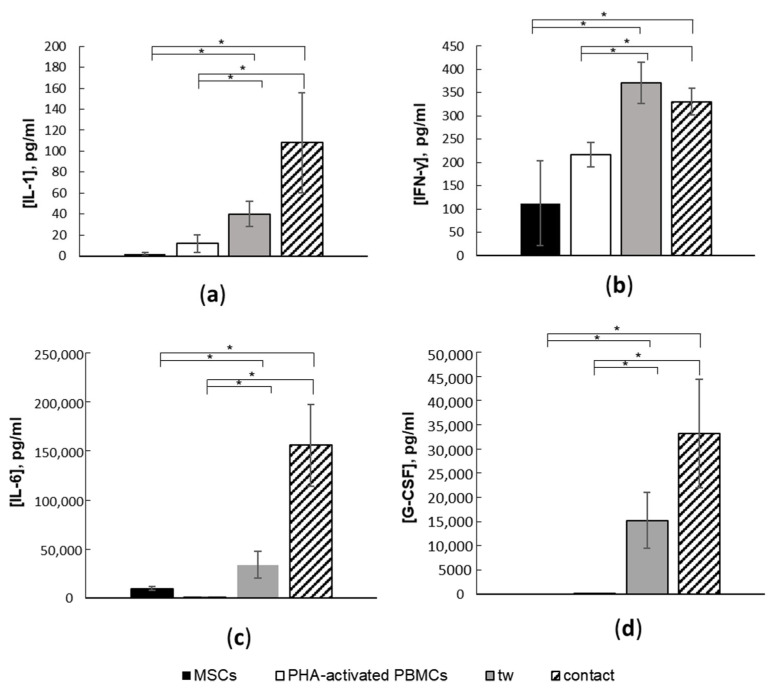
Suppression of PBMC proliferation in the presence of MSCs accompanied by enhanced production of (**a**) IL-1, (**b**) IFN-γ, (**c**) IL-6, and (**d**) G-CSF. Data are shown as mean ± std; *n* = 5, *t*-test, * reliability of differences at *p* < 0.05. Contact refers to contact MSC–PBMC co-culture, tw refers to non-contact co-culture. Five unrelated donors per cell type were used.

Our results indicate that MSCs can skew the production of cytokines when co-cultured with activated PBMCs. The interaction of MSCs with activated PBMCs led to the substantial enhancement of IL-1, IL-6, IL-8, IFN-γ, MCP-1, and G-CSF production in supernatants. Moreover, the concentrations of IL-1(Figure 6a), IL-6 (Figure 6b), and G-CSF (Figure 6c) were significantly higher in contact co-cultures compared to non-contact conditions. On the other hand, the enhancement of IFN-γ secretion in co-cultures did not depend on the presence or absence of direct cell–cell contact between MCSs and activated PBMCs (Figure 6d). In co-cultures, the production of IL-8 and MCP-1 increased more than five-fold, such that their concentration values were above the detection limits (not shown). In this case, the effect of cellular contact between MSCs and activated PBMCs on the IL-8 and MCP-1 release has not been estimated.

**Figure 7:**
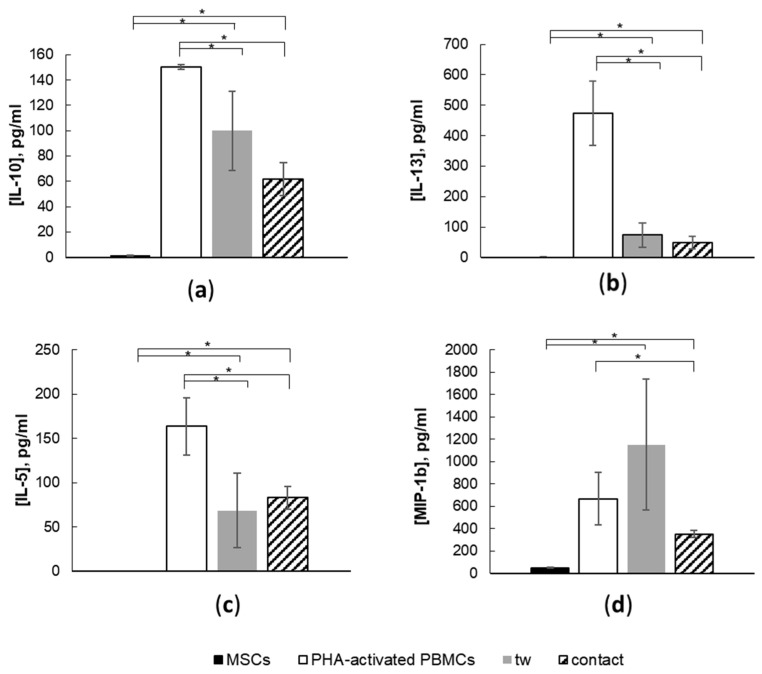
Production of (**a**) IL-10, (**b**) IL-13, (**c**) IL-5, and (**d**) MIP-1b by activated PBMCs is inhibited in contact and non-contact (tw) co-cultures with MSCs. Data are shown as mean ± std; *n* = 5, *t*-test, * reliability of differences at *p* < 0.05. Contact refers to contact MSC–PBMC co-culture, tw refers to non-contact co-culture. Five unrelated donors per cell type were used.

Conversely, the secretion of IL-5, IL-10, IL-13, and MIP-1b was downregulated upon the interaction between activated PBMCs and MSCs (Figure 7). No significant differences were found between concentrations of IL-5 (Figure 7a), IL-10 (Figure 7b), or IL-13 (Figure 7c) in contact and non-contact co-cultures, so the inhibition of their production occurred through contact-independent mechanisms. At the same time, a decreased MIP-1b concentration was observed only in contact co-cultures of MSCs with activated PBMCs.

The amounts of secreted IL-2, IL-4, IL-7, IL-12, IL-17, TNF-alpha, and GM-CSF did not change significantly in the supernatants of co-cultured MSCs and activated PBMCs compared to their monocultures (data not shown).

## 4. Discussion

It has become more and more clear that the therapeutic benefits of MSCs are attributable to multiple immunomodulatory effects that promote the successful healing of damaged tissues [22,23,24,25]. Nevertheless, the complex and multifactorial processes that occur during the interaction of resident or transplanted MSCs with immune cells at sites of acute and chronic inflammation require further analysis.

MSCs have been shown to be capable of modulating the functions of various immune cells through soluble factors secreted in response to the proinflammatory microenvironment [25,26,27]. These observations suggest that MSCs possess the capacity to inhibit inflammatory responses in vivo [28]. Our results also reveal that MSCs possess the ability to inhibit PHA-induced PBMC proliferation, and this effect is accompanied by changes in the expression and secretory profile of MSCs.

Studies to date have shown that in response to the proinflammatory microenvironment, MSCs can secrete molecules such as COX-2, IDO, iNOS, TGF-beta, galectins, PD-L, etc. [3,6,29]. In the present study, the expression of inflammatory mediator genes COX-2, IDO, and iNOS was assessed in MSCs at rest or following co-culture with activated PBMCs. Our results indicate that MSCs express COX-2 constitutively when cultured under resting conditions. As expected, the level of COX-2 expression in MSCs was significantly increased upon interaction with activated PBMCs. Previous reports also revealed that COX-2 production significantly increases in the supernatants following MSC and PBMC interaction in co-culture, and the COX-2 inhibitor indomethacin has been shown to significantly counteract MSC-mediated suppression of PBMC proliferation [30,31]. Increased COX-2 expression in MSCs under the influence of proinflammatory cytokines, aggregation into spheroids, and interaction with scaffolds have also been noted by other researchers [14,32,33,34,35]. In our experiments, we found that the increased COX-2 expression in co-cultured MSCs occurred in a dose-dependent manner that was proportional to the number of PBMCs and did not depend on the presence of cell–cell contact. Since MSCs constitutively express COX-2, and the supernatant from resting MSCs does not suppress PBMC proliferation, it can be assumed that, in this case, alternative inhibition mechanisms may be involved through cross-linking signals from other subpopulations of immune cells. For example, several studies have examined monocytes as essential intermediaries through which MSCs mediate their suppressive effects on T cell proliferation [36].

In other studies, the functional activity of IDO in MSCs was shown to contribute to inhibiting activated PBMC proliferation due to the depletion of tryptophan and the accumulation of kynurenine and its degradation products [35,37,38,39]. We observed that, in contrast to COX-2, MSCs did not constitutively express IDO in monoculture. The induction of IDO expression in MSCs occurred in response to inflammatory signals derived from the microenvironment produced by activated PBMCs. In noncontact co-culture, the IDO expression level in MSCs was directly proportional to the amount of activated PBMCs and therefore to the concentration of cytokines secreted by them. Our observations are consistent with our previous studies and others showing that cytokine production by activated immune cells is correlated with the induction of IDO expression in MSCs [18,37,38]. However, IDO expression levels in MSCs upon contact interaction with activated PBMCs were significantly higher compared to those in non-contact conditions and did not show a correlation with an increased amount of activated PBMCs in co-culture. These findings suggest that only a limited number of PBMCs are able to form adhesive contacts through transmembrane receptors that activate the IDO signaling pathway in MSCs, confirming the importance of contact interactions between MSCs and activated PBMCs for the induction of IDO synthesis. This is also supported by our previous studies, in which direct MSC–T lymphocyte contact via intercellular adhesion molecules (ICAMs) was demonstrated to be essential for the regulation of IDO synthesis in MSCs [18].

Our data reveal the absence of iNOS expression in MSCs both in monoculture conditions and in co-culture with activated PBMCs. Thus, the immunosuppressive functioning of MSCs did not rely on NO activity in our experimental conditions. Other reports also indicate negligible nitrite concentrations in monocultures of human PBMCs and MSCs, as well as in their co-cultures [38]. Some authors attribute the lack of iNOS expression in MSCs under inflammatory stimulation to the high variance in mechanisms of molecular immunosuppression between mammalian species, which can be divided into two groups: human, monkey, and pig MSCs synthesize IDO to suppress immune responses, whereas in rodents this effect is mediated by NO [38,40]. On the other hand, the lack of iNOS expression in MSCs can also be explained by the conditions in which the detection was performed, because the kinetics of regulatory factor accumulation in cells induced by various inflammatory mediators can differ significantly. This is confirmed by the data of Hara et al. (2008) showing that in the presence of a NOS inhibitor, IDO activity in cells increased at the posttranscriptional level, and conversely, exposure to NO inhibited IDO activity [41]. Thus, in our experimental conditions, the lack of iNOS expression in MSCs under interaction with PBMCs might be attributed to the cross-inhibition of iNOS synthesis. Since we detected IDO synthesis in MSCs, the inhibition of the iNOS signaling pathway in MSCs at the same time is an expected event. However, we cannot rule out the possibility that other culture conditions and timing might promote iNOS expression in human MSCs, as we previously observed. For example, we found that MSCs can express iNOS at the transcriptional level when co-cultivated with unstimulated PBMCs [42]. These observations confirm that the induction of IDO synthesis in MSCs is one of the early events that can trigger a cascade of subsequent signaling interactions between different subpopulations of immune cells, causing sequential changes in the cytokine microenvironment of damaged tissue and promoting successful healing.

In the present study, we quantitatively investigated the secretory response of MSCs and activated PBMCs to environmental cues occurring in a co-culture system using intact cultures of MSCs and activated PBMCs as a baseline. A potential weakness of this approach lies in its reductionist formulation, since we focused on the suppression of PHA-induced PBMC proliferation as a functional test for MSC activity, although the immunosuppressive function of MSCs is revealed as a dynamic response to the environment. Nevertheless, we demonstrate here that intact MSCs constitutively secreted high levels of IL-6, IL-8, MCP-1, and COX-2. These data support the assumption that initially intact MSCs exhibit a pro-inflammatory phenotype.

Previous studies have also noted the ability of MSCs to produce high levels of pro-inflammatory cytokines [6,14,43,44]. In a recent study, secretome analysis showed that T cell suppression correlates with the generation of a unique cytokine signature that is upregulated by MSCs or downregulated in responder PBMCs [45]. In this study, we identified cytokine outputs that correlate with the suppression of PBMC proliferation in vitro. The observed upregulation of IL-1, IL-6, IL-8, IFN-γ, MCP-1, and G-CSF indicates a multiple response that may result in the activation of regulatory pathways in various lymphoid and myeloid cells. In contrast, the downregulation of the anti-inflammatory cytokine IL-10, along with IL-13, IL-5, and MIP-1b, may reflect the multifunctional response of PBMCs to the suppressive effect of MSCs. The dependence of IL-1, IL-6, G-CSF, and MIP-1b production on the presence or absence of direct contacts between cells in MSC and PBMC co-cultures indicates the importance of intercellular communication in their regulation.

The complex approach we employed here provides an opportunity to capture the effector pathways of MSCs that are regulated by activated PBMCs. The regulation of IDO is associated with the activation of IFN-γ signaling pathways [46,47]. The interaction of TNF with TNF-R leads to the formation of a multiprotein signaling complex at the cell membrane that triggers a series of intracellular events, resulting in the activation of nuclear transcription factor κB (NF-κB), which regulates the expression of almost 400 genes, including iNOS and COX-2 enzymes [48,49,50,51].

Under our experimental conditions, we observed an increase in the IFN-γ concentration in supernatants of MSCs and activated PBMC co-cultures, accompanied by the induction of IDO synthesis in MSCs, indicating activation of the IFN-γ signaling pathway in MSCs. On the other hand, we found that the TNF-α concentration in supernatants of MSCs and activated PBMC co-cultures did not undergo significant changes across the experiments. These data, in combination with the constitutive expression of COX-2 and the absence of iNOS expression in MSCs, suggest that the TNF-α signaling pathway is not activated in MSCs co-cultured with activated PBMCs under our experimental conditions. Therefore, our results indicate that nitrite and COX-2/PGE2 secretion by MSCs is not responsible for the inhibition of PBMC proliferation. Thus, changes in IFN-γ secretion and IDO synthesis in MSCs may be directly related to the suppression of activated PBMC proliferation. Changes in the concentration of other cytokines may indicate the subsequent modulation of immune cells. These facts seem to be consistent with the normal physiological tissue response to injury that occurs during this period of time.

In recent years, promising approaches aimed at increasing the therapeutic efficacy of MSCs have included primed cytokines, growth factors, hypoxia, pharmaceuticals, biomaterials, and different culture conditions [52]. Our findings, which reveal the upregulation of IL-1, IL-6, IL-8, IFN-γ, MCP-1, and G-CSF in MSC and PBMC co-cultures, highlight a potential area to search for inducers of MSC therapeutic activity. Currently, there is strong evidence that preconditioning with IFN-γ enhances the immunomodulatory properties and therapeutic efficacy of MSCs by promoting the expression of IDO [53,54,55,56,57,58]. On the other hand, it has been reported that stimulation of MSCs through the NF-κB pathway can also induce some synergistic and overlapping functionalities with integrative effects. Several studies demonstrated that preconditioning MSCs with IL-1 enhances their survival, migration, adhesion, chemokine production, and angiogenic activity, as well as the efficacy of MSC transplantation in DSS-induced colitis [55,59,60,61]. Other researchers indicate that MSCs treated with TNF-α possess enhanced angiogenic and osteogenic activity in vitro and in vivo [62,63,64,65]. MCP-1 priming was also shown to enhance the therapeutic effects of MSCs in contact-hypersensitive mice by activating the COX2-PGE2 pathway [66].

Several studies address the potential modulatory effects of G-CSF and IL-6 on MSCs in vitro. Stimulation of MSCs by G-CSF was shown to modulate various pathways, including the metabolism of hyaluronan and migratory capacity, specifically through reduced CD44 expression [67]. MSC treatment with IL-6 resulted in increased production of pro-inflammatory cytokines [68]. The involvement of cell–cell interactions in regulating the inflammatory response supports the idea that in addition to MSC priming through surface adhesion molecules, the route of MSC administration (local or systemic) can determine the effectiveness of MSC-based therapy [28,69,70]. However, the effectiveness of MSCs treated with these molecules still needs to be confirmed by further experimental and clinical evidence.

In conclusion, the results presented here show that stimulation by inflammatory signals and the establishment of adhesive intercellular contacts with target cells are required for MSCs to exhibit immunosuppressive properties. It was demonstrated that MSC-mediated suppression of PBMC proliferation in co-cultures is accompanied by the induction of IDO synthesis in MSCs, and a simultaneous increase in the concentrations of IL-1, IL-6, IL-8, MCP-1, and G-CSF in supernatants. Furthermore, the regulation of IL-1, IL-6, and G-CSF production was accomplished with the involvement of direct cell–cell contact between MSCs and PBMCs. These findings provide new insights into approaches to searching for and identifying potential precondition inducers or their combinations to obtain functionally qualified MSCs for more effective treatment of inflammatory diseases.

## Data Availability

Data available upon request from the authors.
